# Anion-Tunable Properties and Electrochemical Performance of Functionalized Ferrocene Compounds

**DOI:** 10.1038/srep14117

**Published:** 2015-09-16

**Authors:** Lelia Cosimbescu, Xiaoliang Wei, M. Vijayakumar, Wu Xu, Monte L. Helm, Sarah D. Burton, Christina M. Sorensen, Jun Liu, Vincent Sprenkle, Wei Wang

**Affiliations:** 1 Energy and Environment Directorate, Richland, WA 99352, USA; 2 Fundamental and Computation Science Directorate, Richland, WA 99352, USA; 3 National Security Directorate, Richland, WA 99352, USA; 4 Environmental Molecular Sciences Laboratory, Pacific Northwest National Laboratory, Richland, WA 99352, USA

## Abstract

We report a series of ionically modified ferrocene compounds for hybrid lithium-organic non-aqueous redox flow batteries, based on the ferrocene/ferrocenium redox couple as the active catholyte material. Tetraalkylammonium ionic moieties were incorporated into the ferrocene structure, in order to enhance the solubility of the otherwise relatively insoluble ferrocene. The effect of various counter anions of the tetraalkylammonium ionized species appended to the ferrocene, such as bis(trifluoromethanesulfonyl)imide, hexafluorophosphate, perchlorate, tetrafluoroborate, and dicyanamide on the solubility of the ferrocene was investigated. The solution chemistry of the ferrocene species was studied, in order to understand the mechanism of solubility enhancement. Finally, the electrochemical performance of these ionized ferrocene species was evaluated and shown to have excellent cell efficiency and superior cycling stability.

Redox flow batteries (RFBs) are rechargeable electrochemical energy storage devices that utilize the redox chemistry of at least one soluble redox couple for energy conversion between electrical energy and chemical energy^1^,^2^,^3^. The positive and negative electrolytes are stored in two separate reservoirs and pumped through the cell compartments where energy is converted. The cell architecture provides RFBs with a unique virtue of decoupled energy capacity and power output, which gives RFBs tremendous design flexibility to meet the different requirements for energy–driven or power–oriented applications. The excellent modularity and scalability makes RFBs especially suitable for large-scale stationary applications such as grid stabilization and renewable integration. Although great progress has been made in the past years, conventional aqueous RFB systems are generally limited by the water electrolysis on cell voltage and by the low solubility of electro-active materials, resulting in generally low energy density systems (less than 25 Wh L^−1^ in practical applications)^4^,^5^. In contrast, non-aqueous electrolytes offer a wider electrochemical window. The main advantage of adopting non-aqueous electrolytes in flow batteries is to achieve higher cell voltage aiming at high energy density storage. Organic compounds are considered as electroactive materials due to the tremendous diversity of the active species and redox mechanisms, and the possibility of incorporating multiple electron transfers via functionality control in the molecular design. In addition, the structure can be synthetically tailored to improve the solubility which in turn results in a higher system energy density^6^,^7^. Therefore, non-aqueous RFBs have attracted considerable attention recently and a number of redox chemistries have been proposed and investigated^1^,^8^.

Among these efforts, we implemented a novel concept: a hybrid lithium-organic non-aqueous RFB (LORFB) that used the Li metal as the anode and an organic electroactive material as the cathode^9^,^10^,^11^. In a synergistic form of RFB and Li-ion battery, this cell design takes advantage of the high energy density of the Li metal and the extremely low redox potential of the Li/Li^+^ couple to harvest a high cell voltage. This LORFB concept was first illustrated by using a structurally tailored anthraquinone (AQ) catholyte, which produced an energy efficiency of 82% and a discharge energy density of ~25 Wh L^−1^ using a static cell^12^. However, although improved from almost zero to 0.25 M in the electrolyte, the solubility of the modified AQ was still low, limiting the energy density that could be harvested from the system. Also, many other reported nonaqueous RFBs were characterized at the electroactive material concentrations of no more than 0.1 M^13^,^14^,^15^,^16^,^17^. Such low concentrations generally result in low energy density RFB systems that cannot fully compete with the aqueous RFB counterparts. Therefore, there is a compelling need to search for organic electroactive candidates having higher redox potentials and solubilities.

Here we report a new LORFB based on the ferrocene/ferrocenium (i.e. Fc/Fc^+^) redox couple as the catholyte material hence abbreviated as the Li|Fc system. Ferrocene-containing compounds, mostly polymers, have been extensively investigated in solid-state Li-ion batteries due to the relatively high redox potential of the ferrocene (~3.4 V versus Li/Li^+^) and stable redox characteristics^6^,^7^. Yu *et al.* published two reports showing impressive performance from ferrocene–based Li batteries, in a different architecture, though in one case, it is not clear whether the ferrocene is functionalized or not^18^,^19^. However, the use of the pristine ferrocene as an active species in flow battery is limited by its low solubility in the supporting electrolytes (e.g. a saturation concentration of 0.04 M in the electrolyte of 1.2 M LiTFSI in an organic solvent mixture). Therefore, a polar tetraalkylammonium ionic moiety was incorporated into the ferrocene structure (i.e. ionized Fc compounds) in an attempt to improve the solubility of the chromophore in polar supporting electrolytes. We investigate the effect of a series of counter anions (A^−^) affixed to the tetraalkylammonium group on properties such as solubility, melting point, bond energies and cell performance. The counterion containing analogs synthesized and studied include bis(trifluoromethanesulfonyl)imide (TFSI^−^), hexafluorophosphate (PF_6_^−^), perchlorate (ClO_4_^−^), tetrafluoroborate (BF_4_^−^), and dicyanamide (N(CN)_2_^−^). The solution chemistry of these ionized Fc electrolytes was studied using nuclear magnetic resonance (NMR) analysis and density functional theory (DFT) to understand the mechanism of solubility enhancement. Finally, the electrochemical performance of these ionic-Fc compounds in their corresponding Li|Fc flow battery was evaluated to demonstrate excellent cell efficiency and superior cycling stability.

## Results and Discussion

### Synthesis of Ionic-Fc analogs

**Figure 1:**
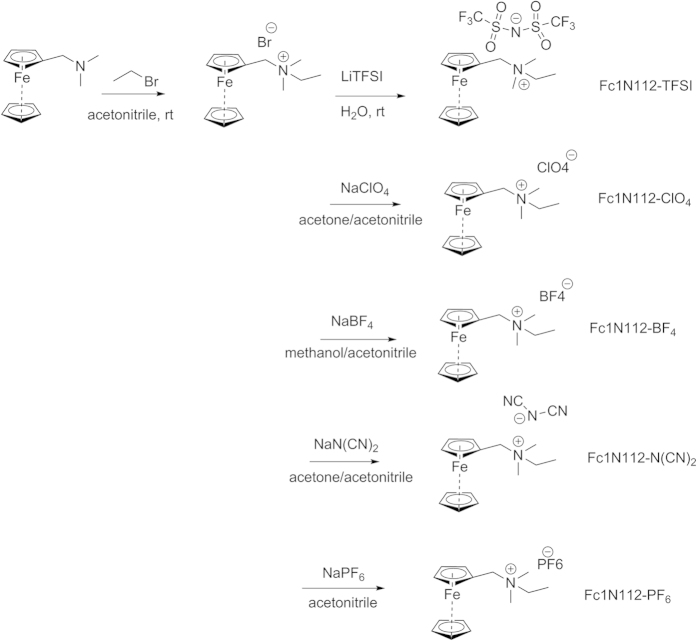
Synthesis of the ionic-Fc analogs bearing different counter anions such as TFSI^−^, ClO_4_^−^, BF_4_^−^, N(CN)_2_^−^, and PF_6_^−^.

According to Fig. 1, all the ionic ferrocene compounds were synthesized from a common bromide salt intermediate (Fc1N112-Br) that was synthesized from commercially available N,N-dimethyl(aminomethyl)ferrocene (Fc1N11) via nucleophilic substitution with bromoethane in acetonitrile. The subsequent ionic Fcs were prepared in decent yields via an ion exchange reaction of the bromide intermediate with the lithium or sodium salt of the desired counter anions including TFSI^−^, ClO_4_^−^, BF_4_^−^, N(CN)_2_^−^, and PF_6_^−^. The ion exchange reactions were performed in an organic, polar, anhydrous solvent, with the exception of the TFSI^−^ and N(CN)_2_^−^ analogs which were performed in de-ionized water. Due to the stability of LiTFSI in water, the ease of removing the LiBr salt byproduct, and ease of isolation of the final product in near quantitative yield, water was the perfect solvent for this synthesis. In the case of the dicyanamide analog, the sodium dicyanamide salt was too insoluble in a suitable organic solvent, therefore the reaction was performed in water, followed by extraction with dichloromethane. The other ion exchange reactions were performed in anhydrous organic solvents such as acetonitrile, methanol or acetone because the starting lithium or sodium salts were too hygroscopic and/or prone to hydrolysis and decomposition in water (for BF_4_^−^ and PF_6_^−^).

### Solubility study

The catholyte used in the Li|Fc system contains an ionic-Fc and the corresponding lithium salt (LiA where A is the counter anion) in a carbonate mixture solvent of ethylene carbonate (EC)/propylene carbonate (PC)/ethyl methyl carbonate (EMC) at a weight ratio of 4:1:5. The solubility of the ionic-Fc analogs was evaluated first in the EC/PC/EMC solvent system, followed by the more relevant 1.2 M LiA supporting electrolytes in EC/PC/EMC as they were the actual medium in the Li|Ionic-Fc cells. The results are summarized in Table 1.

The pristine ferrocene has a low solubility in both solvent and electrolyte, reaching saturation at 0.2 M in EC/PC/EMC and at only 0.04 M in 1.2 M LiTFSI in EC/PC/EMC. It is obvious that incorporation of the ionic pendants resulted in a significant increase in the solubility of all the Fc chromophores, although they exhibited quite different solubility values. In EC/PC/EMC, the Fc1N112-N(CN)_2_ had the highest solubility of 2.08 M, followed by 1.73 M for Fc1N112-TFSI, 1.71 M for Fc1N112-PF_6_, 0.63 M for Fc1N112-ClO_4_, and 0.40 M for Fc1N112-BF_4_, respectively. The solubilities of all ionic-Fcs in the corresponding supporting electrolytes (1.2 M LiA in EC/PC/EMC) were reduced compared with those in the solvent: 0.85 M for Fc1N112-TFSI, 0.65 M for Fc1N112-PF_6_, 0.38 M for Fc1N112-ClO_4_, and 0.38 M for Fc1N112-BF_4_, respectively. The solubility of Fc1N112-N(CN)_2_ in the electrolyte of Li N(CN)_2_ in EC/PC/EMC was not measured due to the lack of a commercial source of Li N(CN)_2_. As previously stated, the solubility of the electroactive materials in the electrolyte is critically important for a RFB system to achieve high energy density delivery. The solubility improvement will result in a significant increase in the system energy density of the Li|Ionic-Fc flow battery.

The goal of this work is not only to determine the most soluble candidate of the Ionic-Fc series for non-aqueous RFB application, but also to understand the solvation mechanism and factors that lead to the solubility increase of the Ionic-Fc molecules. In a simplified model, the solvation phenomenon can occur in two different ways: (1) the cation and anion fragments of the Ionic-Fc are separated by the solvent molecules; or (2) the Ionic-Fc molecule as a whole is surrounded by the solvent molecules such that the entire pair is solvated. One study by Fox *et al.*^20^ suggests that the ionic liquid is not strongly interacting with the solvent (acetonitrile or PC), but rather stays closely associated in solution. Similar work by the same group shows a strong aggregation behavior of ionic liquids dependent on the solvent used^21^. Therefore, in depth studies of density functional theory (DFT) calculations and solution NMR characterizations were performed in order to gain a comprehensive understanding of the solvation mechanism.

### DFT Study

The solubility of an ionic salt critically depends on two competing interactions namely the ion–solvent and cation–anion (*i.e.* ion pair) interactions which transpires as solvation phenomena. The solubility of the Fc1N112^+^ - anion salt in EC/PC/EMC solvent mixture clearly showed an explicit counter anion dependent behavior, indicating varying degree of cation-anion interaction strengths (i.e. affinity between specific anion and Fc quaternary ammonium cation). To verify this hypothesis, the characteristics of the Fc1N112 cation-anion bonds in terms of affinity (i.e. electronic binding energy ΔE) and ion-pair distance (i.e. shortest distance between Fc1N112^+^ and anion) was analyzed using DFT calculations. The geometry of the Fc1N112^+^ cation-anion pairs was optimized under open-shell electron configuration using the hybrid B3LYP function with a slater type TZ2P all electron basis set implemented in the ADF 2013 package. Although such a single pair gas phase calculation excluded the highly correlated ion- solvent mixture interactions and temperature effects, it qualitatively captures the relative affinity between specific anion and Fc quaternary ammonium cation.

**Figure 2:**
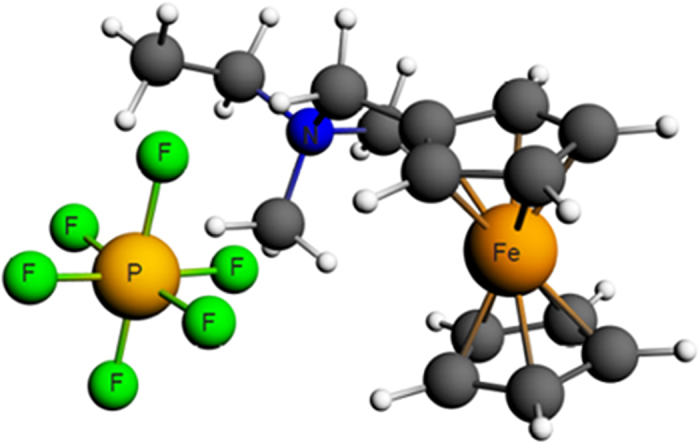
Geometry-optimized structures for Fc1N112-PF_6_ single pair molecule using DFT theory with B3LYP functional and TZ2P basis electron set. The carbon and hydrogen atoms are represented as grey and white spheres respectively.

Figure 2 shows the fully optimized geometry of the Fc1N112-PF_6_ molecule. To extract the correct bond distance between these complex molecules, the electron density profile was calculated on the optimized structure. For the unsymmetrical Fc1N112^+^ cation the positive charge density is mainly concentrated on the nitrogen atom, while for the PF_6_^−^ anion the negative charge density is nearly equally distributed among the fluorine atoms. Therefore, the typical cation-anion bond distance should be the shortest distance between the cation nitrogen atom and the anion in the molecule. This was verified by the good agreement between the calculated bond distance of 4.08 Å and the single crystal structure experimental value of 4.20 Å (Supplemental Information) of the Fc1N112-TFSI molecule. Then, the cation-anion affinity (i.e. electronic energy differences ΔE) was calculated by the difference between the electronic bonding energy of the ion pair and the isolated cation/anion, *i.e.* Δ*E* = (*ion* − *pair*)_*BE*_ − (*cation*)_*BE*_ − (*anion*)_*BE*_.

**Figure 3:**
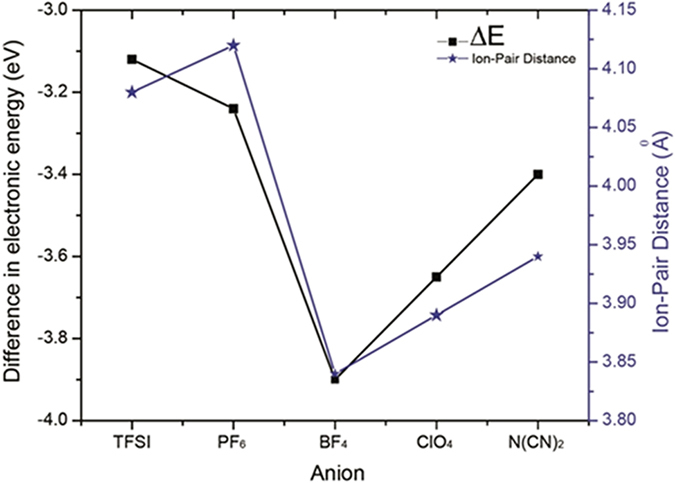
The bonding energy and bonding distance for different anion molecules calculated from the DFT optimized geometry.

Figure 3 shows the single pair electronic energy differences ΔE and ion-pair distance between the Fc1N112^+^ cation and various anions. The BF_4_^−^ and ClO_4_^−^ based ion pair molecules show large bonding energies along with shorter bonding distances, indicating a lower propensity for solvation. On the other hand, the TFSI^−^ and PF_6_^−^ anions show relatively lower affinity and positions at relatively longer distances with the Fc1N112^+^ cation, suggesting anions more likely to be solvated. The N(CN)_2_^−^ anion has an intermediate affinity and ion-pair distance which lies between the above two groups of anions. In general, the trend of the calculated affinities and ion-pair distances of these Ionic-Fcs matches well with that of their solubility data, which provides a potential qualitative explanation of the solubility difference among the various Ionic-Fcs. However, the Fc1N112-N(CN)_2_ exhibits an extremely high solubility in the carbonate solvent mixture although it has intermediate bonding energy and bonding distance. The reasons for this anomalous behavior need to be studied further. Based on this study, we confirm that the high solubility of Fc1N112-TFSI and Fc1N112-PF_6_ could be a consequence of the relatively weaker affinity between the TFSI^−^ or PF_6_^−^ anion with the Fc1N112^+^ cation, which allows for the solvent insertion between the ion pair. Our DFT calculations suggest a solvation mechanism where the ion-pair retention in solution is less likely and the ions are surrounded by solvent molecules and subsequently experience higher solubility. The DFT calculations do not take into account the ion-solvent mixture interactions as the system would become very complex (our solvent has three components) and results difficult to interpret, however, we do not exclude the possibility of this solvation mechanism.

### NMR studies

The NMR studies aim to explore solvent-ion interactions in a 0.2 M solution of EC/PC/EMC with a weight ratio of 4:1:5. In order to decouple the potential interaction of TMS with the concentrated ferrocene-based analogs solutions, we conducted co-axial ^1^H NMR with an external tetramethylsilane (TMS) reference. At this concentration, we followed the response of the solvent peaks to the different ionic-Fc environments.

**Figure 4:**
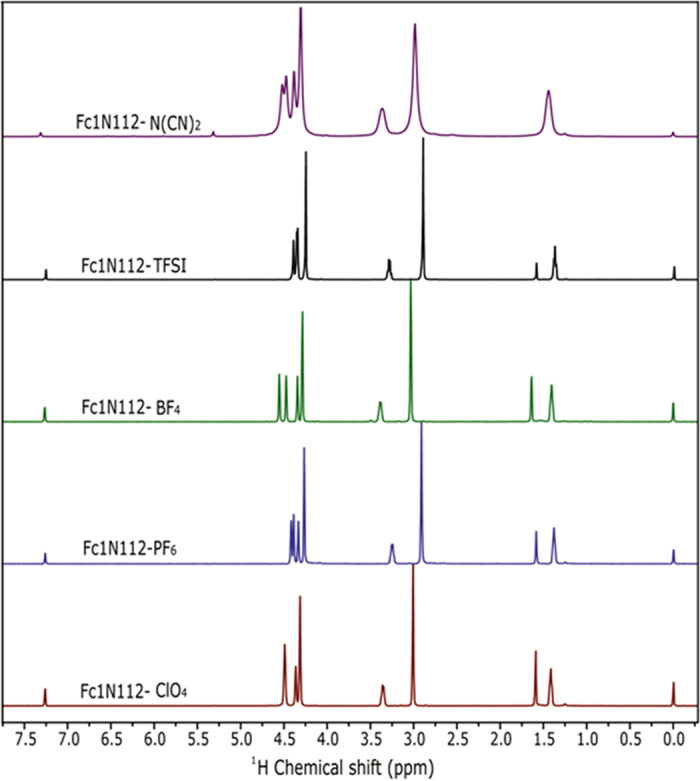
Stacked ^1^H NMR spectra of the ionic-Fc analogs at low concentration in deuterated chloroform. The spectra were referenced to internal TMS standard.

The substrates were dissolved in EC/PC/EMC (4:1:5) at concentrations of 0.2 M with an external TMS reference (co-axial tube) in a neat solvent system and the mixtures were subjected to ^1^H NMR tests. The proton shifts of the solvent remain the same for EC, PC and EMC regardless of the ionic-Fc present, which indicates that in the context of NMR scale (*i.e.*, milliseconds to seconds) and at a relatively low concentration (0.2 M) the chemical environment of the solvent molecules does not change. Although a higher concentration would have been beneficial to gain more conclusive insights, we chose the concentration of 0.2 M because it was the highest concentration at which *all* analogs were soluble. This experiment suggests that no fixed molecular bond or interaction between any of the solvent molecules and the ionized molecules occurs at this concentration. Furthermore, this indicates that the solvation mechanism at low concentrations is one in which the entire Ionic-Fc is surrounded by the solvent molecules, and there is no preferential interaction with any part of the Ionic-Fc. If there are long range interactions between the solvent and the ionic pair, it is not detectable by NMR. Although all counter ions have different degrees of polarizability, the interactions may be too small to be detected by NMR. This supports the hypothesis that the Ionic-Fc remains generally bound, not allowing preferential solvent interactions with the cation or anion. The extent of bonding between the anion and the cation of each analog differs with the nature of the respective anion and its polarizability and it is reflected in the low concentration ^1^H NMR in deuterated chloroform, Fig. 4. Slight shifts and change in splitting patterns are observed in each cation, as a response to the different environments of various counter anions.

Another explanation of the different solvation lies in the shape and size of the overall ionic pair and how the different counter anions affect the crystal lattice. This can be easily probed by comparing melting points of all the analogs. Indeed, the melting point trend (see Table 1) in all analogs but PF6^−^, which was not measured, follows the same trend observed in the solubility of the respective species: DCA^−^ has the lowest melting point at 81.5 °C, followed closely by TFSI^−^ at 85 °C, followed by BF4^−^ at 148.5 °C then closely by ClO4^−^ at 153 °C.

In conclusion the solvation behavior appears to be a complex phenomenon and several factors and mechanisms may play in concert. DFT calculations show an energy and ion-pair distance trend that agrees with experimental solvation data; however, they do not take into account the various solvents surrounding the molecule. Melting point data suggests that the energy of the crystal lattice plays a role in the solvation of the Ionic-Fcs and this data is also in agreement with the solubility data. Co-axial ^1^H NMR however, given the 0.2 M concentration limitation and the inherent inability of detect long-range molecular interactions, suggests that solvents do not preferentially interact with any of the Ionic-Fcs.

### Electrochemical performance

**Figure 5:**
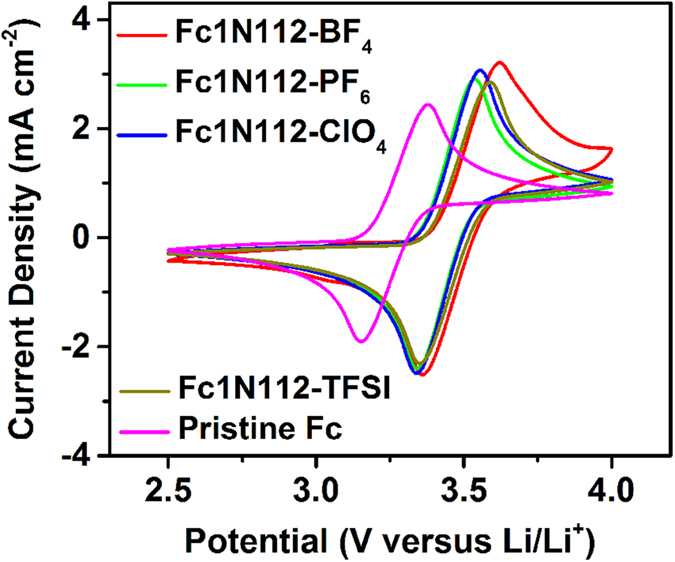
CV curves of 0.05 M of the Ionic-Fcs and the pristine Fc in 1.2 M LiA supporting electrolytes in EC/PC/EMC (4:1:5 by wt.) at a scan rate of 50 mV s^−1^. Fc was measured in 1.2 M LiTFSI electrolyte.

The electrochemical behavior of the four Ionic-Fcs was studied as the catholyte materials in the hybrid Li|Ionic-Fc batteries (the N(CN)_2_-analog was not evaluated due to the unavailability of LiN(CN)_2_). Figure 5 shows the cyclic voltammetry (CV) curves of the four Ionic-Fcs and the pristine Fc in 1.2 M corresponding LiA electrolytes in EC/PC/EMC (4:1:5, by wt.), where the LiTFSI salt was used for the unmodified Fc.

The pristine Fc exhibited the oxidation and reduction peaks at 3.38 V and 3.15 V versus Li/Li^+^, respectively, yielding an average redox potential of 3.26 V. Incorporation of the ionic pendants shifts the Fc species to higher redox potentials by ~0.2 V, which is considered to be closely associated with the electron withdrawing effect of the quaternary ammonium functionality. The counter anions in the ionic arms have a relatively minor effect on the electrochemical properties of the ionic-Fc analogues, resulting in similar electrochemical reversibility and redox potentials. The redox reactions of Fc1N112-ClO_4_ and Fc1N112-PF_6_ are slightly more reversible than the other two. The four ionic-Fcs, exhibit an average redox potential of 3.49 V for Fc1N112-BF_4_, 3.47 V for Fc1N112-TFSI, 3.45 V for Fc1N112-ClO_4_, and 3.44 V for Fc1N112-PF_6_, versus Li/Li^+^ as listed in Table 1. The positive shift of the redox potential and the improved solubility function in synergy to increase the energy density of the Li|Ionic-Fc cells. For example, the Li|Ionic-Fc employing the Fc1N112-TFSI as the catholyte has a theoretical energy density of 79 Wh L^−1^ that is twice as high as that of the aqueous mixed-acid VRB system^22^.

The cell performance of the Li|Ionic-Fc batteries was investigated in static cell configurations with the Ionic-Fc concentrations at their nominal solubilities in 1.2 M LiA in EC/PC/EMC. The coin cells were galvanostatically cycled in the constant current mode at a current density of 1.5 mAcm^−2^ between 3 and 4 V. Reliable protection of the solid electrolyte interphase (SEI) on the Li surface is crucial for lithium metal-based rechargeable batteries, because continuous dendrite growth during repeated charge/discharge cycles may cause internal short of the cell which is one of the major battery safety hazards^23^,^24^. Fluoroethylene carbonate (FEC) has been recognized as an effective electrolyte additive for Li, carbon and Si anodes to form good quality SEI layers and then enhance battery performance^25^. Therefore, a 5 wt% of FEC was used in each of the Ionic-Fc catholytes.

**Figure 6:**
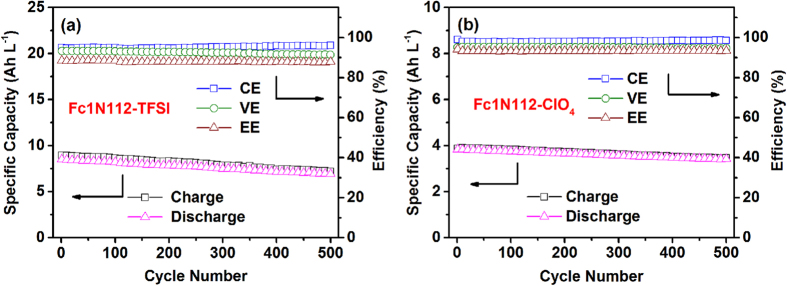
The cycling efficiency and specific volumetric capacity at 1.5 mA cm^−2^: (a) the Li|Fc1N112-TFSI static cell; and (b) the Li|Fc1N112-ClO_4_ static cell.

Among the four Ionic-Fc analogs, the static cells of Li|Fc1N112-TFSI and Li|Fc1N112-ClO_4_ systems produced excellent long-term performance over extended cycling. Figure 6a,b show the cycling efficiency and the specific volumetric capacity of the Li|Fc1N112-TFSI and the Li|Fc1N112-ClO_4_ static cells, respectively. Both Li|Fc1N112-TFSI and Li|Fc1N112-ClO_4_ static cells exhibited remarkably stable performance and high cell efficiencies which were maintained over 500 cycles. The Li|Fc1N112-TFSI cell produced 95% Coulombic efficiency (CE), 92% voltaic efficiency (VE) and 88% energy efficiency (EE), while the Li|Fc1N112-ClO_4_ cell produced even higher efficiencies with 98% CE, 95% VE and 93% EE. These cell efficiencies are significantly higher than those reported in most non-aqueous redox chemistries^26^,^27^,^28^. The Li|Fc1N112-TFSI cell delivered an energy density of ~28 Wh L^−1^ that is comparable to practical mixed-acid VRB systems^22^. The initial discharge energy density of the Li|Fc1N112-ClO_4_ cell was ~13 Wh L^−1^ due to the relatively low concentration of the Fc1N112-ClO_4_ in its electrolyte, but it is still comparable to the aqueous iron-vanadium system. The lower CE of the Li|Fc1N112-TFSI cell was due to the higher cycling capacity (~8 vs ~3.7 Ah L^−1^) leading to longer per cycle time and more self-discharge reactions when tested at the same current density. Excellent capacity retention was achieved for both systems, and an average capacity loss of 0.04% and 0.02% per cycle was observed for the Li|Fc1N112-TFSI and Li|Fc1N112-ClO_4_ cells, respectively, throughout the 500 cycles. Such a performance exceeds many other reported non-aqueous RFB systems which suffer from serious degradation^15^,^16^,^29^. The excellent cyclability is attributed to the stabilized SEI layer contributing to the effective suppression of the self-discharge reactions between the charged species at the anode and cathode compartments.

In contrast, the Fc1N112-PF_6_ and the Fc1N112-BF_4_ produced poor cyclability and the coin cells using these two compounds failed after only a few cycles. The failure mechanism is speculated to be due to the poor solubility of the charged species, *i.e.*, the Ionic-ferrocenium salts, or more specifically PF_6_^−^-Fc^+^1N^+^112-PF_6_^−^ and BF_4_^−^-Fc^+^1N^+^112-BF_4_^−^, in the electrolytes. In an effort to prove this hypothesis, all of the coin cells were disassembled and examined after cycling. Solid precipitation was observed in the Li|Fc1N112-PF_6_ and Li|Fc1N112-BF_4_ cells but not in the Li|Fc1N112-TFSI and Li|Fc1N112-ClO_4_ cells. Further research is warranted to identify the source of the precipitation and elucidate the battery failure mechanism.

## Conclusions

The transition from aqueous to non-aqueous battery systems provides great benefits but it is not without challenges. Appropriate active materials can achieve higher energy and power densities and efficiencies. The use of organic electroactive species offers both opportunities and challenges for the design and synthesis of novel materials with tunable solubilities, redox potentials and activities. Our present work was focused on understanding the effect of the counter anion on the solubility and performance of a series of ferrocene containing ionic species. The data demonstrates a wide range of solubility for the various anions studied N(CN)_2_^−^ > TFSI^−^ > PF_6_^−^ > ClO_4_^−^ > BF_4_^−^ from 2.08 M to 0.40 M in EC:PC:EMC (4:1:5), while the electrochemical performance shows a different trend. Both Li|Fc1N112-TFSI and Li|Fc1N112-ClO_4_ static cells exhibited remarkably stable performance and high cell efficiencies which were maintained over 500 cycles. The Li|Fc1N112-TFSI cell produced 95% CE, 92% VE and 88% EE, while the Li|Fc1N112-ClO_4_ cell produced even higher efficiencies with 98% CE, 95% VE and 93% EE. In contrast, the Fc1N112-PF_6_ and the Fc1N112-BF_4_ produced poor cyclability and the coin cells using these two compounds failed after only a few cycles. In conclusion, a high solubility of the active species is required but not sufficient in assessing battery performance. The solubility and stability of the charged species formed as a result of redox chemistry will need to also be assessed. Although the electrochemical performance of several ferrocene ionic species is quite remarkable, there is still a need for better understanding of long-term stability of organic species in their charged and neutral states to optimize performance.

## Methods

### Synthesis

All reagents and starting materials were purchased from Sigma-Aldrich and used as received unless noted otherwise. All reactions were run in an inert atmosphere in oven dried glassware. Experimental details can be found in the Supporting Information. Mass spectrometry: A one milligram of sample was dissolved into 2 mL of methanol. The sample was electrosprayed at 3 microliters per minute into a LTQ-Orbitrap (Thermo Fischer Scientific. The electrospray emitter (150 micrometers OD and 20 micrometers ID at tip) was custom made using the protocol described by Kelly *et al*.^30^. Data acquisition was an average of 100 scans with a FT mass resolution setting of 30000. X-Ray: Single crystals of Fc1N112-TFSI were grown by slow solvent evaporation of a saturated solution of methylene chloride. A suitable crystal was selected and mounted on a nylon loop with Paraton-n oil on a Bruker APEX-II CCD diffractometer. The crystal was kept at 120 K during data collection. Using Olex2^31^, the structure was solved with the olex2.solve structure solution program using Charge Flipping and refined with the ShelXL^32^ refinement package using Least Squares minimization. The structure is shown in Figure S7 while crystal data, bond distances and angles are shown in Tables S1-S2 in the SI.

### NMR measurements

^1^H and ^13^C NMR were performed on a Varian 500 MHz NMR spectrometer equipped with a Z axis-gradient 5 mm HCN probe. ^1^H NMR co-axial experiments of concentrated samples (0.2 M) were externally referenced to tetramethylsilane (TMS) to avoid TMS interaction with ferrocene, while regular low concentration ^1^H NMR spectra were referenced to internal TMS.

### DFT calculations

Quantum chemistry calculations were carried out using the *Amsterdam Density Functional* (ADF-2013) program. The hybrid-GGA based Becke, three-parameter, Lee-Yang-Parr DFT function (B3LYP) with Grimme dispersion correction (D3) and TZ2P (triple Z, 2 polarization functions, all electron) basis set function is used. All basis sets and preceding calculations were done with the Slater type functional implemented in the ADF program for both geometry optimization and bonding energy calculation.

### CV tests

Lithium salts (LiTFSI, LiPF_6_, LiBF_4_, LiClO_4_), carbonate solvents (EC, PC, EMC) and the FEC additive in battery grade were ordered from BASF Battery Materials (Independence, OH) and used as received. The solvent mixtures and electrolytes were prepared inside an argon-filled MBraun glove box (Stratham, NH) with H_2_O and O_2_ levels both less than 1 ppm. The CV curves were run between 2.5 and 4 V on a CHI760D Potentiostat (CH Instruments, Austin, TX) at a scan rate of 50 mV s^−1^ with a 2-mm diameter Pt working electrode, a Li metal counter electrode and a Li metal reference electrode. The three-electrode cells for the CV tests were placed inside the MBraun glove box.

### Static cell tests

The performance of Li|Ionic-Fc batteries was tested in 2325-type coin cells. The cell configuration employed a Li foil (99.9%, Alfa Aesar) as the anode, a 2.5-mm-thick graphite felt (SGL Carbon Group, Germany) as the cathode substrate, and a polyethylene-based microporous separator in between. The active area of the coin cell was 1.6 cm^2^. The graphite felt was saturated with an Ionic-Fc catholyte solution (~0.2 mL) with the concentration at its nominal solubility in a 1.2 M LiA supporting electrolyte in EC/PC/EMC (4:1:5, by wt.). The catholyte contained a 5 wt% FEC to protect the SEI on the Li surface. The galvanostatic charging/discharging cycling of the coin cells was performed on an Arbin BT-2000 battery tester (Arbin Instruments, College Station, TX) at 1.5 mA cm^−2^ in a voltage range of 3–4 V.

## Additional Information

**How to cite this article**: Cosimbescu, L. *et al.* Anion-Tunable Properties and Electrochemical Performance of Functionalized Ferrocene Compounds. *Sci. Rep.* **5**, 14117; doi: 10.1038/srep14117 (2015).

## Supplementary Material

Supplementary Information

## Figures and Tables

**Table 1:** Solubility and theoretical energy density of the ionic-Fc species.

Ionic-Fc Counteranion (A^−^)	**TFSI^−^**	**PF_6_^−^**	**BF_4_^−^**	**ClO_4_^−^**	**N(CN)_2_^−^**
In EC/PC/EMC 4:1:5 (M)	1.73	1.71	0.40	0.63	2.08
In 1.2 M LiA in EC/PC/EMC (M)	0.85	0.65	0.38	0.38	*
Mp (°C)	85–86.5	**	148.5–150	153–156	81.5
Redox Potential (V versus Li/Li^+^)	3.47	3.44	3.49	3.45	*
Theoretical Energy Density (Wh L^−1^)	79	60	35	35	*

^*^Not tested due to in-availability of LiN(CN)_2_.

^**^Not measured due to instability of Fc-PF_6_.
