# Supplementary material for: Anion-Tunable Properties and Electrochemical Performance of Functionalized Ferrocene Compounds
Source: Sci Rep. 2015 Sep 16;5:14117. doi: 10.1038/srep14117 (PMC4571638; doi:10.1038/srep14117)
Supplement: Supplementary Information [file srep14117-s1.pdf]

Supplementary Information for

Anion-Tunable Properties and Electrochemical Performance of Functionalized Ferrocene Compounds

Lelia Cosimbescu,[a,] Xiaoliang Wei,[a] Vijayakumar Murugesan,[b] Wu Xu,[a] Monte L. Helm,[b] Sarah D. Burton,[c] Christina, M. Sorensen,[d] Jun Liu,[a] Vincent Sprenkle,[a] Wei Wang[a,*]*

[a] Energy and Environment Directorate, [b] Fundamental and Computation Science Directorate, [c] National Security Directorate, [d] Environmental Molecular Sciences Laboratory, Pacific Northwest National Laboratory, Richland, WA 99352, USA

*****CORRESPONDING AUTHORS: Lelia Cosimbescu, Wei Wang.

Chemical modification of the ferrocene structure.

Synthesis of N-(ferrocenylmethyl)-N,N-dimethyl-N-ethylammonium bromide (Fc1N112-Br). To a solution of (dimethylaminomethyl)ferrocene (19.4 g, 79.8 mmol, 1eq) in acetonitrile (150 mL) was added bromoethane (6.2 mL, 83.0 mmol, 1.05eq) dropwise. The mixture was stirred at room temperature overnight to ensure completion of the reaction. The formed precipitate was isolated by filtration and washed with diethyl ether, to yield 20.5 g of orange product. The filtrate was concentrated, and the precipitated second crop was isolated by filtration to yield an additional 5.9 g of product. The total yield was 26.4 g, 94%. ^{1}H NMR (500 MHz, $CDCl_3$) δ (ppm): 4.84 (s, 2H), 4.53 (s, 2H), 4.34 (s, 2H), 4.30 (s, 4H), 3.58 (q, *J* = 7.2 Hz, 2H), 3.22 (s, 6H), 1.42 (t, *J* = 7.1 Hz, 3H). ^{13}C NMR (125 MHz, $CDCl_3$) δ (ppm): 71.99, 70.43, 69.40, 64.92, 58.42, 48.93, 8.54. HRMS (ESI) calculated for $C_{15}H_{22}NFe^+$ m/z = 272.1101, found m/z = 272.1086; calculated for Br m/z = at 78.9183, found m/z = 78.9203; calculated for total m/z = 351.0285, found m/z = 351.0289.

Synthesis of N-(ferrocenylmethyl)-N,N-dimethyl-N-ethylammonium bis(trifluoro methanesulfonyl)imide (Fc1N112-TFSI). To a solution of Fc1N112-Br (15 g, 42.6 mmol, 1 eq) in deionized water (100 mL) was added slowly a solution of LiTFSI (12.84 g, 44.7 mmol, 1.05 eq) in deionized water (50 mL). Ion exchange reaction occurred instantaneously, indicated by the immediate formation of a yellow precipitate. As the mixture became too thick additional water was added to aid stirring. The mixture was stirred at room temperature for an additional 2 hours, and the precipitate was isolated by filtration and washed with deionized water (200 mL). The final product, Fc1N112-TFSI, was obtained as a bright yellow powder (23.0 g, 97%), which was dried under vacuum for 24 hours, transferred into an argon-filled glove box and used without further purification. Melting point: 85-86.5 °C. ^{1}H NMR (500 MHz, $CDCl_3$) δ (ppm):

4.47 – 4.31 (m, 6H), 4.25 (s, 5H), 3.28 (q, J = 7.0 Hz, 2H), 2.89 (s, 6H), 1.37 (t, J = 7.0 Hz, 3H). ^{13}C NMR (125 MHz, $CDCl_3$) δ (ppm): 119.83 (quartet, $J_{C\text{-}F}$ = 318.7 Hz), 118.57, 71.81, 71.53, 70.81, 69.48, 65.77, 58.87, 48.66, 8.10. HRMS (ESI) calculated for $C_{15}H_{22}NFe^+$ m/z = 272.1101, found m/z = 272.1093; calculated for $C_2F_6S_2O_4N^-$ m/z = at 279.9173, found m/z = 279.9233; calculated for total m/z = 552.02745, found m/z = 552.0326.

Synthesis of N-(ferrocenylmethyl)-N,N-dimethyl-N-ethylammonium perchlorate (Fc1N112-ClO_4). A solution of sodium perchlorate (1.27 g, 10.3 mmol, 1 eq) in acetone (20 mL) was added to a solution of Fc1N112-Br (3.65 g, 10.3 mmol, 1 eq) in acetonitrile (20 mL) via cannula, with stirring under Ar. The mixture was allowed to stir overnight at room temperature, and then the solvents were removed on a rotary evaporator under reduced pressure. The product was dissolved in dichloromethane (100 mL) and the insoluble NaBr was removed by filtration. The process was repeated once more, with a smaller volume of dichloromethane (20 mL). The filtrate was concentrated and dried to yield 3.54 g (92%) of orange-brown solid which was stored in a glove box and used without further purification. Melting point: 153-156 °C. ^{1}H NMR (500 MHz, $CDCl_3$) δ (ppm): 4.49 (s, 4H), 4.38 (s, 2H), 4.31 (s, 5H), 3.355 (q, J = 5 Hz, 2H), 3.01 (s, 6H), 1.41 (t, J = 5Hz, 3H). ^{13}C NMR (125 MHz, $CDCl_3$) δ (ppm): 72.99, 71.96, 69.97, 69.13, 68.91, 63.49, 58.08, 48.37, 7.80. HRMS (ESI) calculated for $C_{15}H_{22}NFe^+$ m/z = 272.1101, found m/z = 272.1089; calculated for ClO_4 m/z = 98.9485, found m/z = 98.9506; calculated for total m/z = 371.0586, found m/z = 371.0649.

Synthesis of N-(ferrocenylmethyl)-N,N-dimethyl-N-ethylammonium tetrafluoroborate (Fc1N112-BF_4). A solution of sodium tetrafluoroborate (1.50 g, 12.8 mmol, 1 eq) in methanol (30 mL) was added to a solution of Fc1N112-Br (4.52 g, 12.8 mmol, 1 eq) in acetonitrile (25 mL) via cannula, with stirring under Ar. The mixture was allowed to stir overnight, and then the

solvents were removed under reduced pressure. Dichloromethane (50 mL) was added and the insoluble solid (NaBr) was removed by filtration. The filtrate was concentrated to a solid residue and the process was repeated with 20 mL dichloromethane. The filtrate was concentrated to dryness, to yield 4.5 g of a mustard solid (97%), which was stored in the glove box and used without further purification. Melting point: 148.5-150 °C. ^{1}H NMR (500 MHz, $CDCl_3$) δ (ppm): 4.55 (s, 2H), 4.47 (s, 2H), 4.34 (s, 2H), 4.29 (s, 5H), 3.385 (q, *J* = 7.2 Hz, 2H), 3.03 (s, 6H), 1.41 (t, *J* = 7.1 Hz, 3H). ^{13}C NMR (125 MHz, $CDCl_3$) δ (ppm): 72.02, 70.57, 69.53, 65.18, 58.50, 48.68, 8.32. HRMS (ESI) calculated for $C_{15}H_{22}NFe^{+}$ m/z = 272.1101, found m/z = 272. 1091; calculated for BF_4 m/z = 87.0029, found m/z = 87.0048; Calculated for total m/z = 359.113, found m/z = 359.1139.

Synthesis of N-(ferrocenylmethyl)-N,N-dimethyl-N-ethylammonium dicyanamide (Fc1N112-N(CN)$_2$). A solution of sodium dicyanamide (1.19 g, 13.4 mmol, 1.1 eq) in water (20 mL) was added slowly to a solution of Fc1N112-Br (4.30 g, 12.2 mmol, 1 eq) in water (25 mL). The resulting mixture was allowed to stir at 40-50°C for 3 hours and then at room temperature overnight. The aqueous solution was extracted with dichloromethane (3 × 30 mL), and the combined organic extracts were washed with deionized water (2 × 20 mL), then dried over anhydrous Na_2SO_4 and finally filtered. The solvent was removed under reduced pressure to yield 2.4 g of an orange-brown solid (58%) which was stored in the glove box and used without further purification. Melting point: 78.5-81.5 °C. ^{1}H NMR (500 MHz, $CDCl_3$) δ (ppm): 4.52 (s, 2H), 4.47 (s, 2H), 4.38 (s, 2H), 4.31 (s, 5H), 3.36 (s, broad, 2H), 2.98 (s, 6H), 1.44 (s, broad, 3H). ^{13}C NMR (125 MHz, $CDCl_3$) δ (ppm): 120.1, 71.94, 71.35, 70.96, 69.68, 65.94, 59.02, 48.98, 8.46. HRMS (ESI) calculated for $C_{15}H_{22}NFe^{+}$ m/z = 272.1101, found m/z = 272.1088;

calculated for $N(CN)_2$ m/z = 66.0092, found m/z = 66.0108; Calculated for total m/z =338.1193, found m/z = 338.1196.

Synthesis of N-(ferrocenylmethyl)-N,N-dimethyl-N-ethylammonium hexafluorophosphate (Fc1N112-PF_6). A solution of sodium hexafluorophosphate (1.74 g, 10.1 mmol, 1 eq) in acetonitrile (30 mL) was added to a solution of Fc1N112-Br (3.55 g, 10.1 mmol, 1 eq) in acetonitrile (25 mL) via cannula, with stirring under Ar. The resulting mixture was allowed to stir overnight then the solvent was removed under reduced pressure. Dichloromethane (50 mL) was added to the residue and the insoluble solids were filtered. The filtrate was concentrated to dryness and the process was repeated with another portion of dichloromethane (20 mL) to yield 3.79 g of the mustard-brown solid (89%) which was washed with 50 mL of hexanes. The hexafluorophosphate salt appeared to be the least stable of all analogs during the quick handling in ambient conditions, as indicated by the darkening of the dichloromethane solution. The product was stored in the glove box and used without further purification. Melting point was not recorded due to the very unstable nature of this analog. ^{1}H NMR (500 MHz, $CDCl_3$) δ (ppm): 4.42 (s, 2H), 4.39 (s, 2H), 4.33 (s, 2H), 4.27 (s, 5H), 3.25 (q, J = 10 Hz, 2H), 2.91 (s, 6H), 1.38 (t, J = 7.2 Hz, 3H). ^{13}C NMR (125 MHz, $CDCl_3$) δ (ppm): 72.04, 71.75, 70.85, 69.72, 65.48, 58.62, 48.66, 8.06. HRMS (ESI) calculated for $C_{15}H_{22}NFe^+$ m/z = 272.1101, found m/z = 272. 1086; calculated for PF_6 m/z = 144.9641, found m/z = 144.9665; Calculated for total m/z = 417.0742, found m/z =417.0751 .

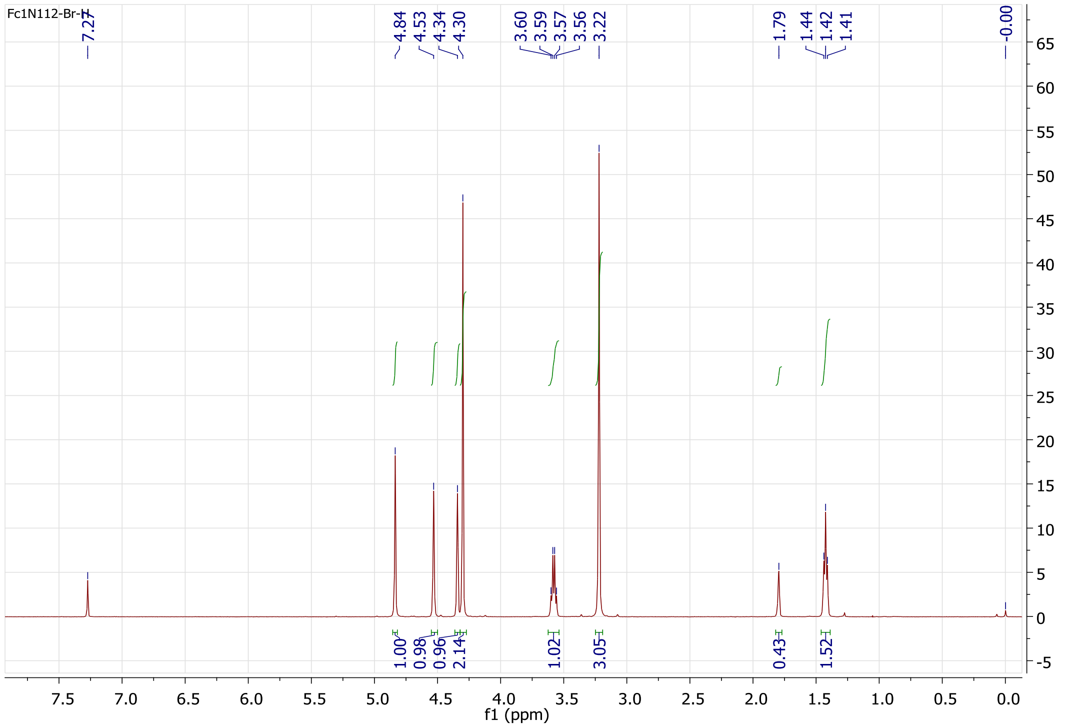

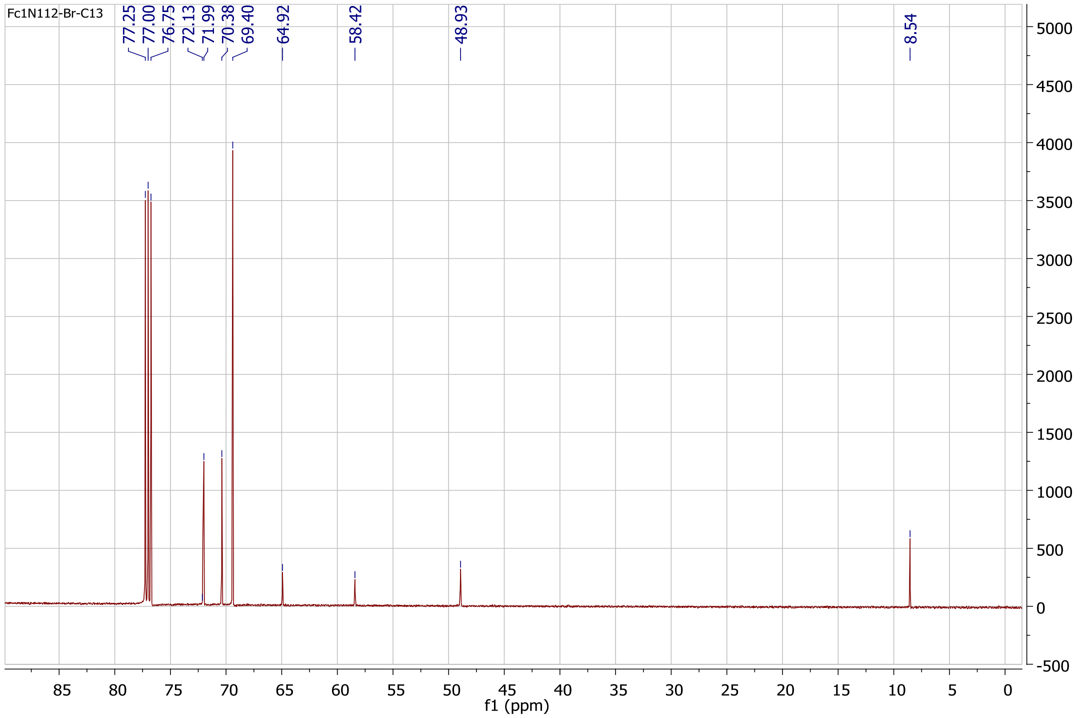

Supplementary Figure S1. ^{1}H and ^{13}C NMR spectra of Fc1N112-Br.

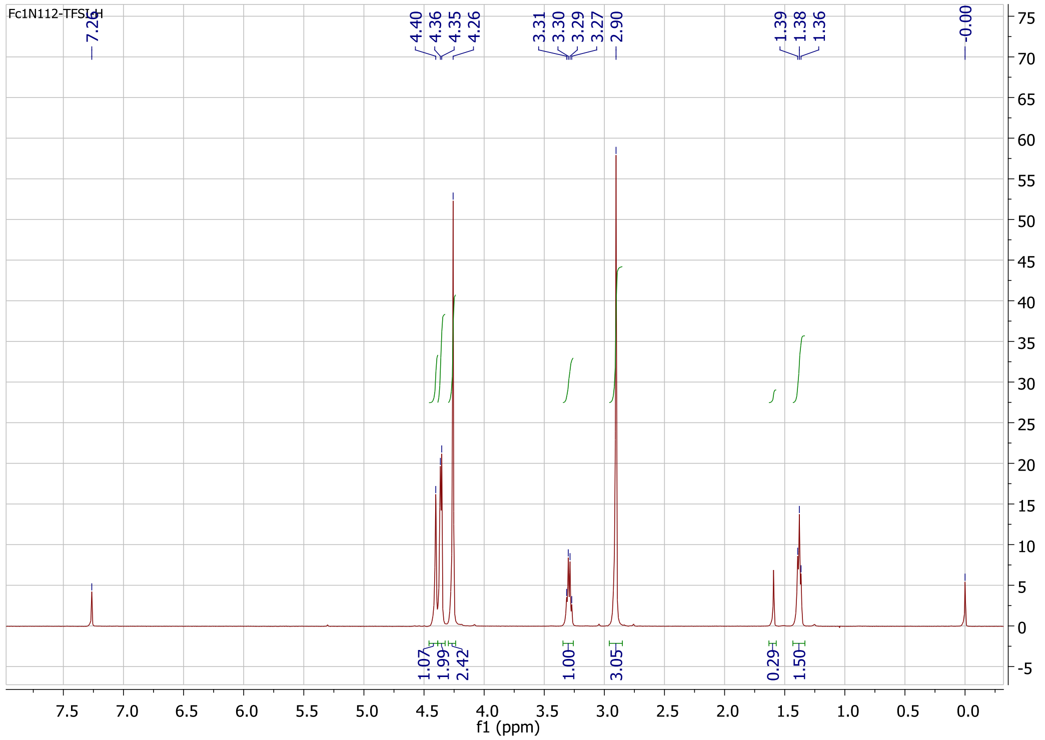

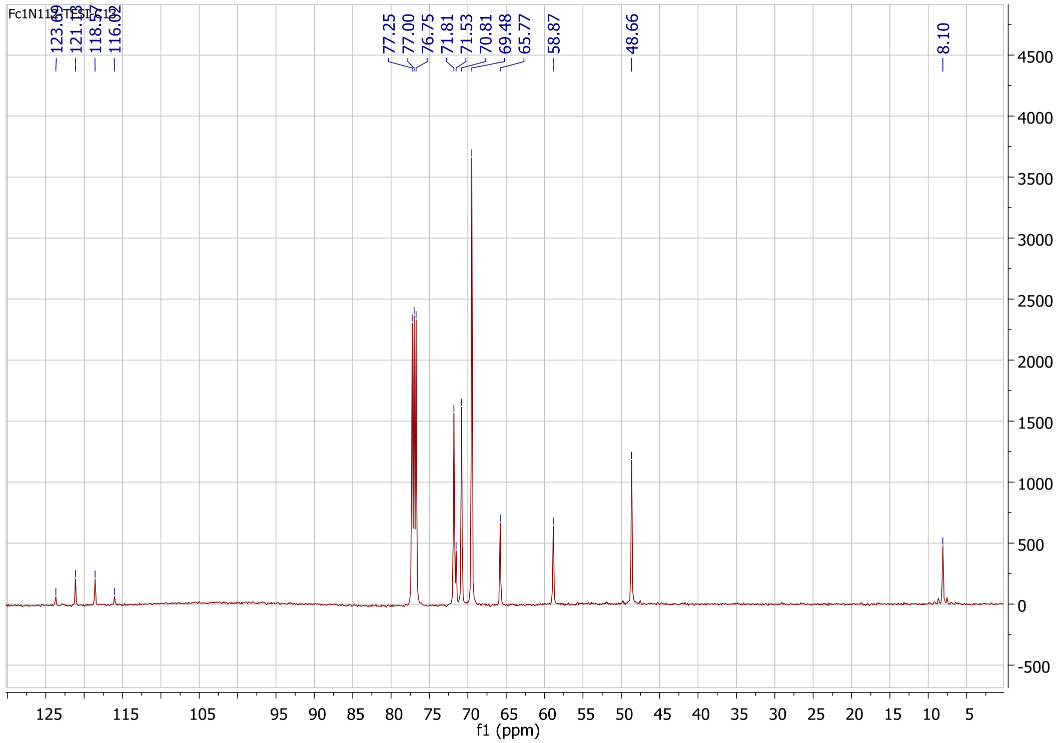

Supplementary Figure S2. ^{1}H and ^{13}C NMR spectra of Fc1N112-TFSI.

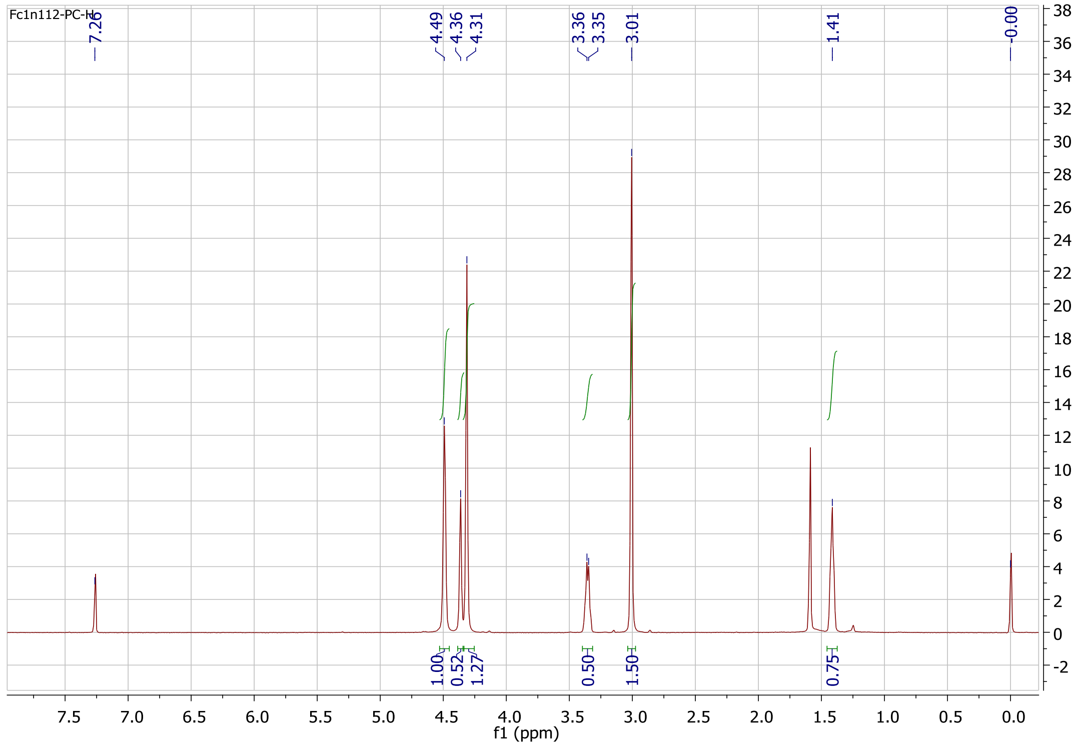

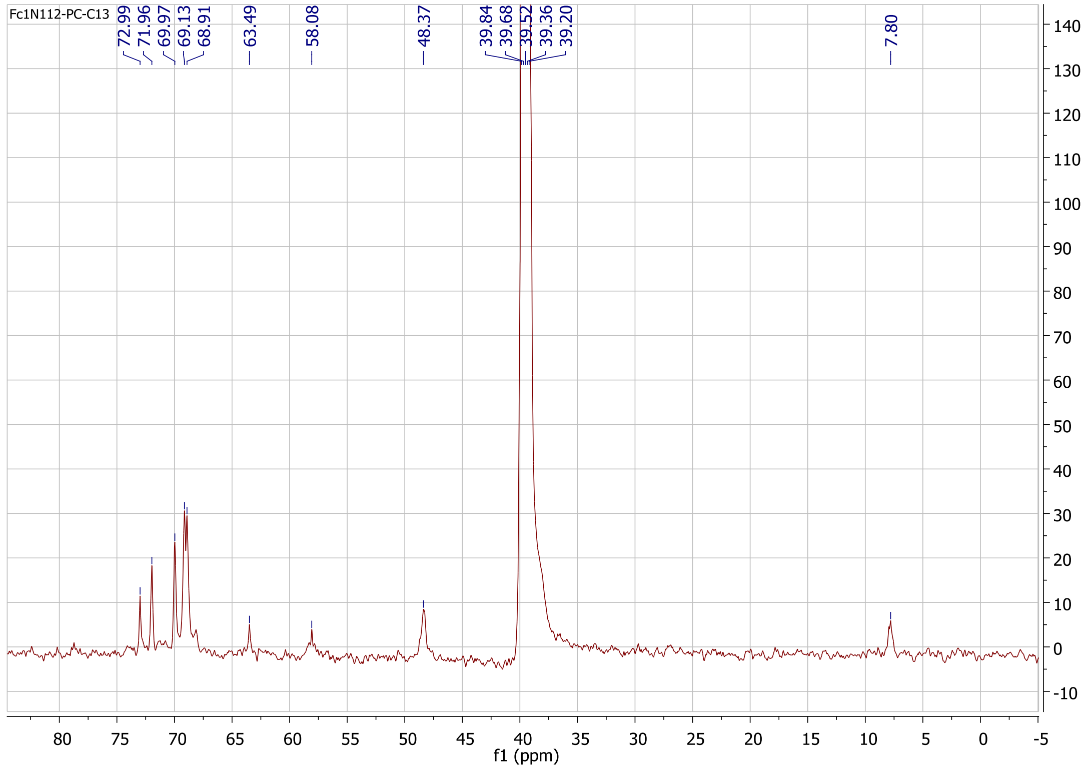

Supplementary Figure S3. ^{1}H and ^{13}C NMR spectra of Fc1N112-ClO_4.

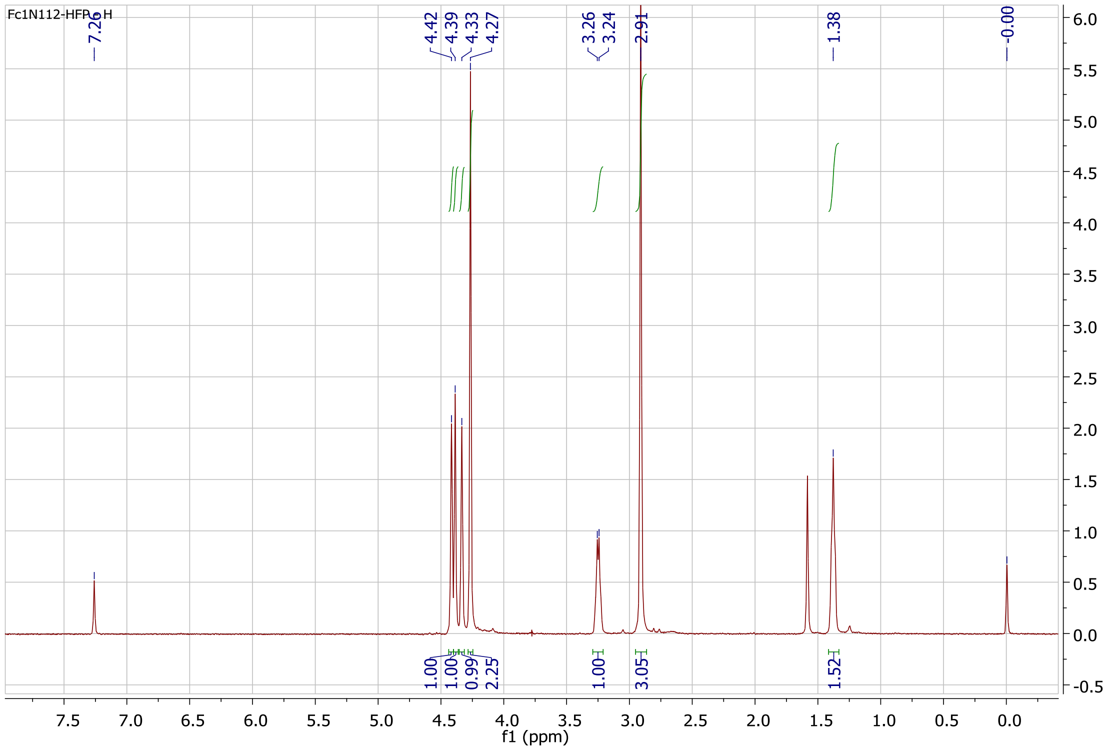

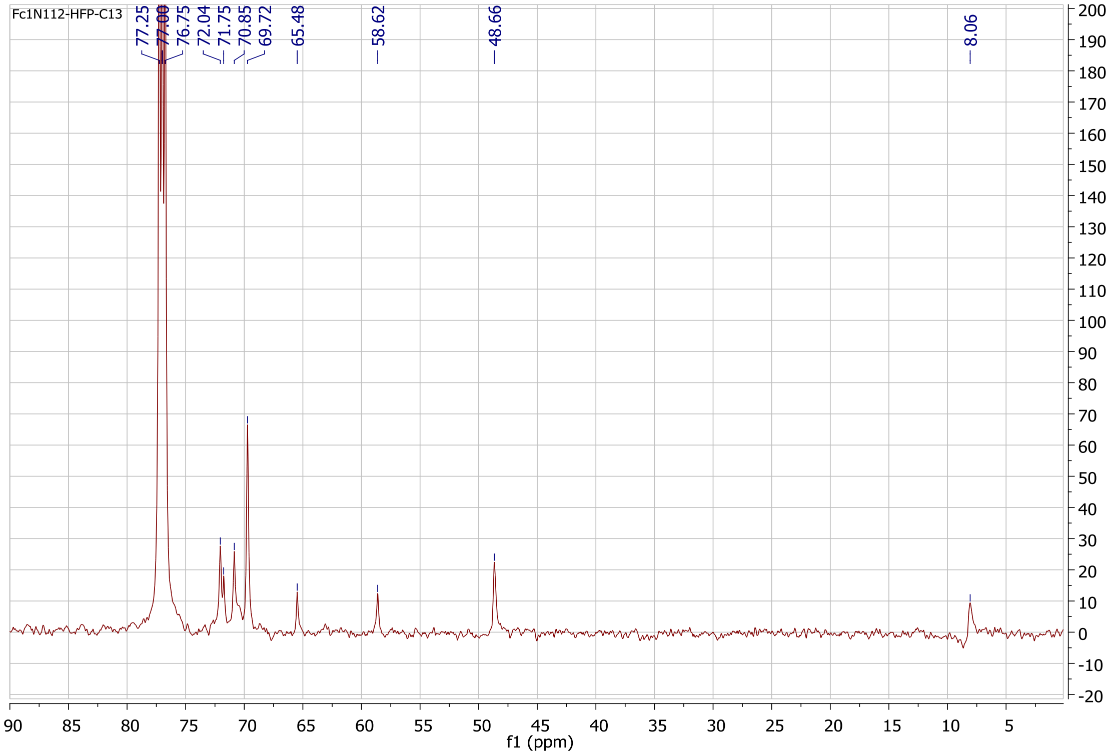

Supplementary Figure S4. ^{1}H and ^{13}C NMR spectra of Fc1N112-PF_6.

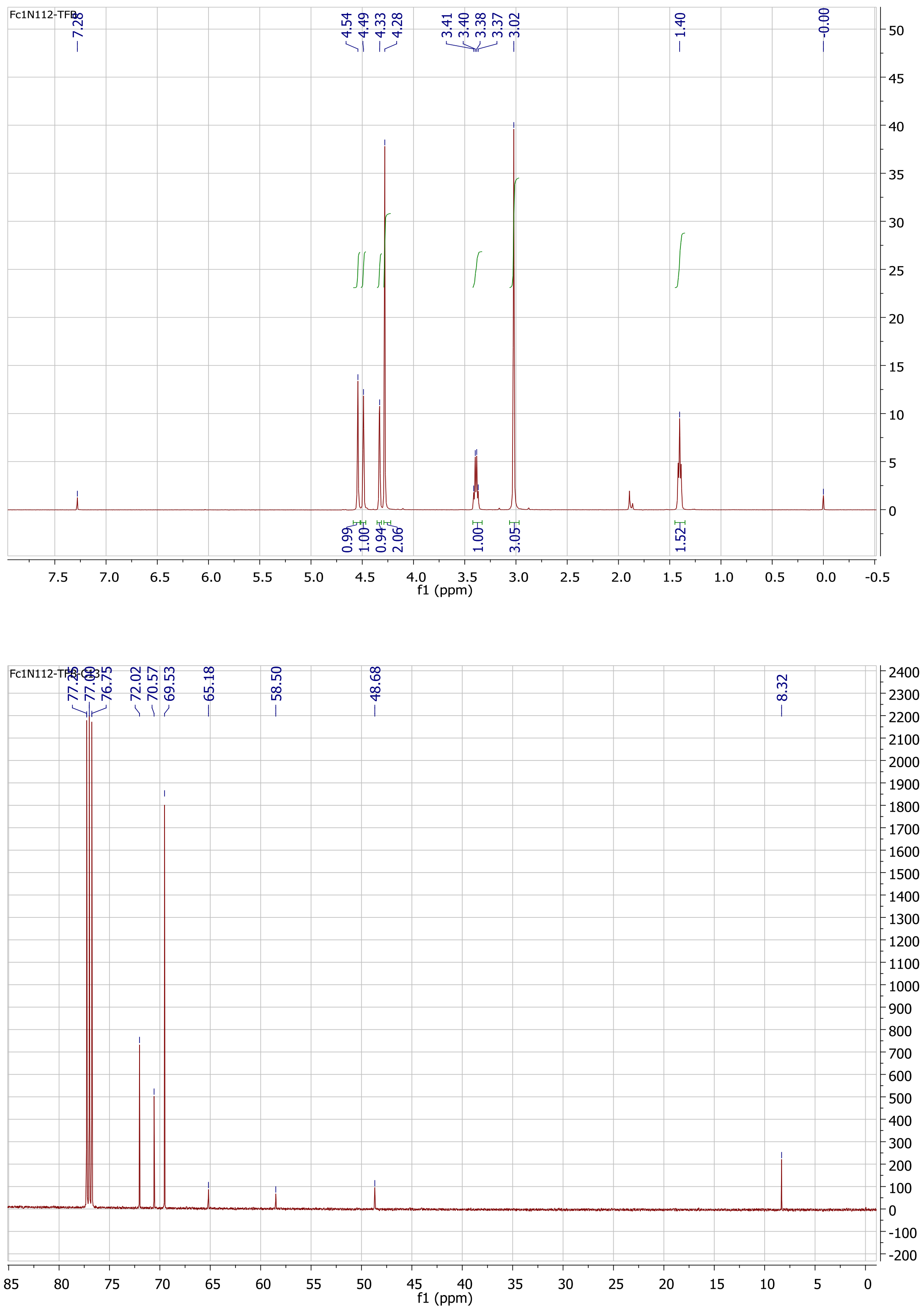

Supplementary Figure S5. ^{1}H and ^{13}C NMR spectra of Fc1N112-BF_4.

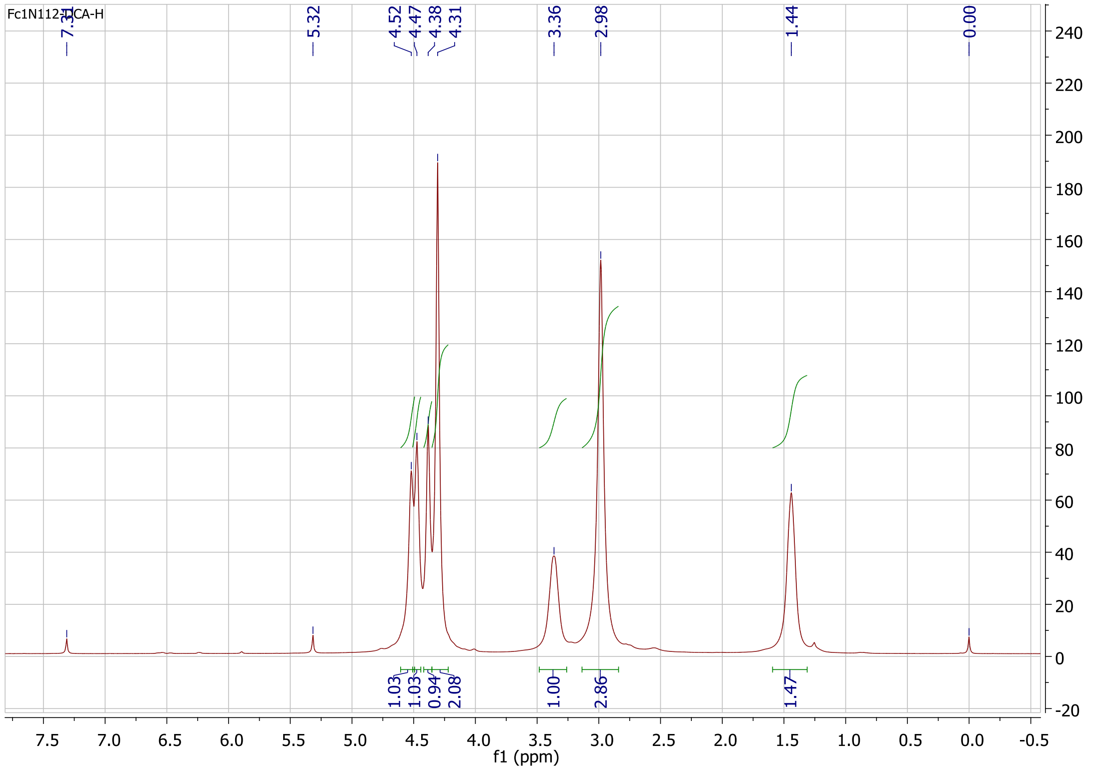

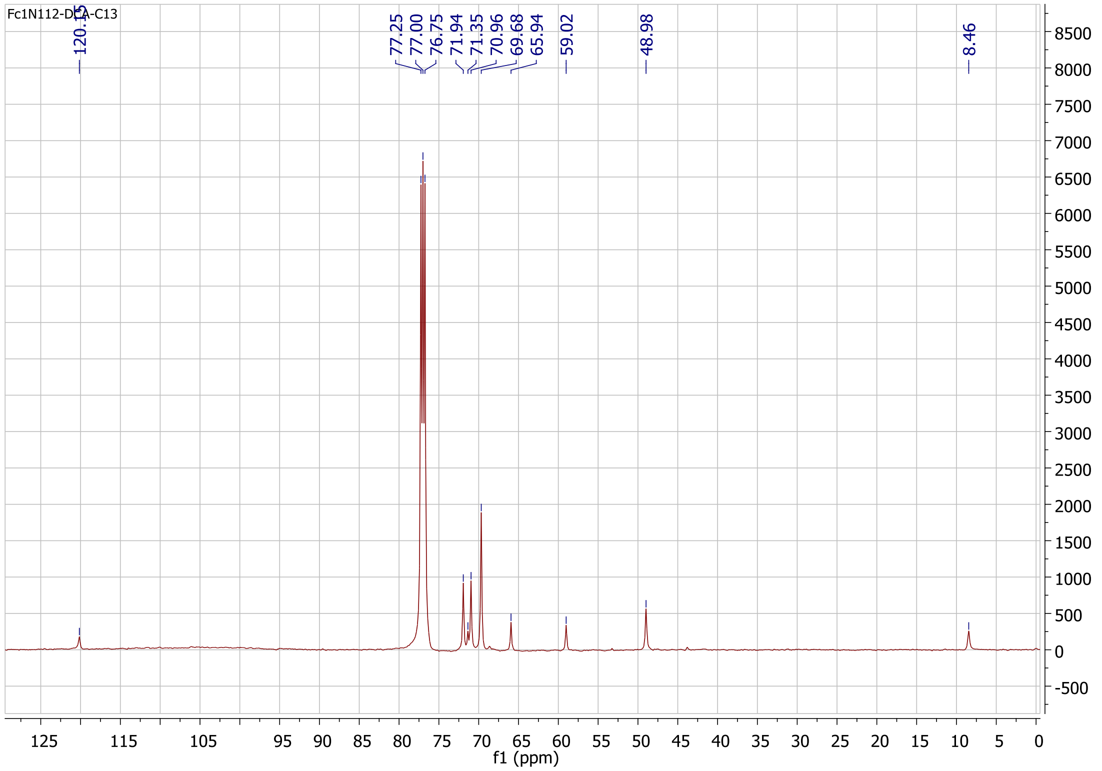

Supplementary Figure S6. ^{1}H and ^{13}C NMR spectra of Fc1N112-$N(CN)_2$.

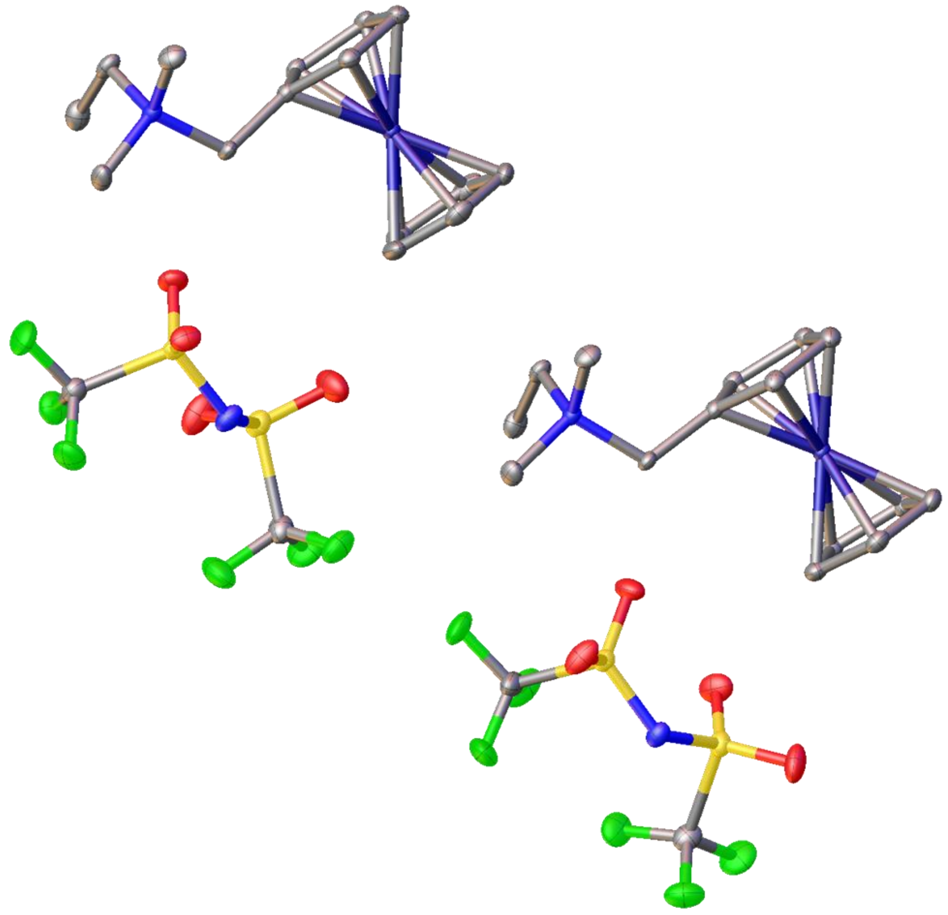

Supplementary Figure S7: X-ray crystal structure of the Fc1N112-TFSI analog. Thermal ellipsoids are shown at the 50% probability level.

Supplementary Table S1. Single crystal X-ray data for **Fc1N112-TFSI**

Identification code	helm174_0m
Empirical formula	$C_{17}H_{22}F_6FeN_2O_4S_2$
Formula weight	552.33
Temperature/K	120
Crystal system	triclinic
Space group	P-1
a/Å	10.5824(6)
b/Å	11.3328(6)
c/Å	19.2369(11)
α/°	99.636(3)
β/°	99.241(3)
γ/°	104.723(3)
Volume/Å^3	2149.3(2)
Z	4
ρ_{calc}mg/mm^3	1.707
m/mm^{-1}	0.975
F(000)	1128.0
Crystal size/mm^3	0.254 × 0.25 × 0.25
Radiation	MoKα (λ = 0.71073)
2Θ range for data collection	2.198 to 63.282°
Index ranges	$-15 \leq h \leq 15$, $-16 \leq k \leq 16$, $-28 \leq l \leq 28$
Reflections collected	64494
Independent reflections	14417 [R_{int} = 0.0283, R_{sigma} = 0.0267]
Data/restraints/parameters	14417/0/583
Goodness-of-fit on F^2	1.037
Final R indexes [I>=2σ (I)]	R_1 = 0.0294, wR_2 = 0.0721
Final R indexes [all data]	R_1 = 0.0379, wR_2 = 0.0770
Largest diff. peak/hole / e Å^{-3}	0.50/-0.41

Supplementary Table S2. Bond lengths (Å) for **Fc1N112-TFSI**

**Atom**	**Atom**	**Length/Å**	**Atom**	**Atom**	**Length/Å**
Fe1	C1	2.0300(11)	C18	C19	1.4319(17)
Fe1	C2	2.0373(11)	C18	C22	1.4322(17)
Fe1	C3	2.0509(12)	C18	C28	1.4888(16)
Fe1	C4	2.0499(12)	C19	C20	1.4195(18)
Fe1	C5	2.0386(12)	C20	C21	1.4234(18)
Fe1	C6	2.0441(12)	C21	C22	1.4238(18)
Fe1	C7	2.0498(12)	C23	C24	1.418(2)
Fe1	C8	2.0462(12)	C23	C27	1.419(2)
Fe1	C9	2.0438(12)	C24	C25	1.419(2)
Fe1	C10	2.0422(12)	C25	C26	1.418(2)
N1	C11	1.5290(14)	C26	C27	1.4153(19)
N1	C12	1.4979(16)	C31	C32	1.5088(19)
N1	C13	1.4958(15)	S1	O1	1.4255(11)
N1	C14	1.5167(15)	S1	O2	1.4302(10)
C1	C2	1.4319(16)	S1	N2	1.5811(11)
C1	C5	1.4320(16)	S1	C16	1.8310(14)
C1	C11	1.4879(16)	S2	O3	1.4249(10)
C2	C3	1.4230(18)	S2	O4	1.4282(10)
C3	C4	1.4225(18)	S2	N2	1.5704(11)
C4	C5	1.4214(18)	S2	C17	1.8223(14)
C6	C7	1.4215(18)	F1	C16	1.3315(15)
C6	C10	1.4254(18)	F2	C16	1.3251(16)
C7	C8	1.4248(19)	F3	C16	1.3229(17)
C8	C9	1.4259(18)	F4	C17	1.3253(19)
C9	C10	1.4241(18)	F5	C17	1.3186(18)
C14	C15	1.5112(18)	F6	C17	1.3305(16)
Fe2	C18	2.0220(11)	S3	O5	1.4271(11)

Fe2	C19	2.0324(12)	S3	O6	1.4267(12)
Fe2	C20	2.0500(12)	S3	N4	1.5856(11)
Fe2	C21	2.0536(12)	S3	C33	1.8287(15)
Fe2	C22	2.0420(12)	S4	O7	1.4315(10)
Fe2	C23	2.0413(13)	S4	O8	1.4325(9)
Fe2	C24	2.0432(13)	S4	N4	1.5670(11)
Fe2	C25	2.0394(13)	S4	C34	1.8337(14)
Fe2	C26	2.0380(12)	F7	C33	1.3283(17)
Fe2	C27	2.0394(13)	F8	C33	1.3264(16)
N3	C28	1.5276(15)	F9	C33	1.3195(17)
N3	C29	1.4994(16)	F10	C34	1.3282(16)
N3	C30	1.4934(16)	F11	C34	1.3221(16)
N3	C31	1.5188(16)	F12	C34	1.3301(15)

Supplementary Table S3. Bond angles (°) for **Fc1N112-TFSI**.

Atom	Atom	Atom	Angle/°	Atom	Atom	Atom	Angle/°
C1	Fe1	C2	41.23(5)	C24	Fe2	C20	162.64(6)
C1	Fe1	C3	68.95(5)	C24	Fe2	C21	126.13(6)
C1	Fe1	C4	68.90(5)	C25	Fe2	C20	125.21(6)
C1	Fe1	C5	41.21(5)	C25	Fe2	C21	108.72(5)
C1	Fe1	C6	106.48(5)	C25	Fe2	C22	121.81(5)
C1	Fe1	C7	121.22(5)	C25	Fe2	C23	68.44(6)
C1	Fe1	C8	157.36(5)	C25	Fe2	C24	40.68(6)
C1	Fe1	C9	159.94(5)	C26	Fe2	C20	107.21(5)
C1	Fe1	C10	122.98(5)	C26	Fe2	C21	121.39(5)
C2	Fe1	C3	40.74(5)	C26	Fe2	C22	156.80(5)
C2	Fe1	C4	68.61(5)	C26	Fe2	C23	68.46(5)
C2	Fe1	C5	69.08(5)	C26	Fe2	C24	68.42(5)

C2	Fe1	C6	120.82(5)
C2	Fe1	C7	156.84(5)
C2	Fe1	C8	160.47(5)
C2	Fe1	C9	123.22(5)
C2	Fe1	C10	106.40(5)
C4	Fe1	C3	40.59(5)
C4	Fe1	C7	124.82(5)
C5	Fe1	C3	68.62(5)
C5	Fe1	C4	40.69(5)
C5	Fe1	C6	123.90(5)
C5	Fe1	C7	107.72(5)
C5	Fe1	C8	121.96(5)
C5	Fe1	C9	157.52(5)
C5	Fe1	C10	160.42(5)
C6	Fe1	C3	156.66(5)
C6	Fe1	C4	161.03(5)
C6	Fe1	C7	40.64(5)
C6	Fe1	C8	68.53(5)
C7	Fe1	C3	161.29(5)
C8	Fe1	C3	124.42(5)
C8	Fe1	C4	108.25(5)
C8	Fe1	C7	40.71(5)
C9	Fe1	C3	107.34(5)
C9	Fe1	C4	121.93(5)
C9	Fe1	C6	68.69(5)
C9	Fe1	C7	68.61(5)
C9	Fe1	C8	40.81(5)
C10	Fe1	C3	121.16(5)
C10	Fe1	C4	157.10(5)
C10	Fe1	C6	40.83(5)
C10	Fe1	C7	68.52(5)
C26	Fe2	C25	40.69(6)
C26	Fe2	C27	40.62(5)
C27	Fe2	C20	120.05(5)
C27	Fe2	C21	155.81(6)
C27	Fe2	C22	161.50(5)
C27	Fe2	C23	40.70(6)
C27	Fe2	C24	68.35(6)
C27	Fe2	C25	68.38(5)
C29	N3	C28	107.37(9)
C29	N3	C31	111.00(10)
C30	N3	C28	109.68(10)
C30	N3	C29	108.44(10)
C30	N3	C31	108.34(10)
C31	N3	C28	111.96(9)
C19	C18	Fe2	69.71(7)
C19	C18	C22	107.59(11)
C19	C18	C28	126.05(11)
C22	C18	Fe2	70.11(7)
C22	C18	C28	126.00(11)
C28	C18	Fe2	120.23(8)
C18	C19	Fe2	68.93(6)
C20	C19	Fe2	70.32(7)
C20	C19	C18	108.16(11)
C19	C20	Fe2	68.99(7)
C19	C20	C21	108.12(11)
C21	C20	Fe2	69.84(7)
C20	C21	Fe2	69.57(7)
C20	C21	C22	108.27(11)
C22	C21	Fe2	69.22(7)
C18	C22	Fe2	68.62(7)
C21	C22	Fe2	70.09(7)

C10	Fe1	C8	68.58(5)	C21	C22	C18	107.86(11)
C10	Fe1	C9	40.79(5)	C24	C23	Fe2	69.76(8)
C12	N1	C11	107.92(9)	C24	C23	C27	107.89(12)
C12	N1	C14	110.46(9)	C27	C23	Fe2	69.58(7)
C13	N1	C11	109.68(9)	C23	C24	Fe2	69.62(7)
C13	N1	C12	108.41(9)	C23	C24	C25	108.00(12)
C13	N1	C14	108.21(9)	C25	C24	Fe2	69.52(7)
C14	N1	C11	112.09(9)	C24	C25	Fe2	69.80(8)
C2	C1	Fe1	69.66(7)	C26	C25	Fe2	69.60(7)
C2	C1	C11	125.64(11)	C26	C25	C24	107.98(12)
C5	C1	Fe1	69.72(7)	C25	C26	Fe2	69.71(7)
C5	C1	C2	107.60(10)	C27	C26	Fe2	69.74(7)
C5	C1	C11	126.69(10)	C27	C26	C25	108.01(12)
C11	C1	Fe1	123.63(8)	C23	C27	Fe2	69.72(8)
C1	C2	Fe1	69.11(6)	C26	C27	Fe2	69.63(7)
C3	C2	Fe1	70.15(7)	C26	C27	C23	108.12(12)
C3	C2	C1	108.03(11)	C18	C28	N3	114.47(9)
C2	C3	Fe1	69.12(7)	C32	C31	N3	114.91(11)
C4	C3	Fe1	69.67(7)	O1	S1	O2	119.22(7)
C4	C3	C2	108.10(11)	O1	S1	N2	116.94(6)
C3	C4	Fe1	69.74(7)	O1	S1	C16	104.93(6)
C5	C4	Fe1	69.23(7)	O2	S1	N2	108.99(6)
C5	C4	C3	108.31(11)	O2	S1	C16	103.22(7)
C1	C5	Fe1	69.07(7)	N2	S1	C16	100.68(6)
C4	C5	Fe1	70.08(7)	O3	S2	O4	117.95(6)
C4	C5	C1	107.97(11)	O3	S2	N2	115.85(6)
C7	C6	Fe1	69.90(7)	O3	S2	C17	104.28(7)
C7	C6	C10	108.03(11)	O4	S2	N2	108.67(6)
C10	C6	Fe1	69.52(7)	O4	S2	C17	103.43(7)
C6	C7	Fe1	69.46(7)	N2	S2	C17	104.92(6)
C6	C7	C8	108.01(11)	S2	N2	S1	124.86(7)

C8	C7	Fe1	69.51(7)	F1	C16	S1	108.70(10)
C7	C8	Fe1	69.78(7)	F2	C16	S1	110.85(9)
C7	C8	C9	108.06(11)	F2	C16	F1	108.07(11)
C9	C8	Fe1	69.50(7)	F3	C16	S1	112.10(9)
C8	C9	Fe1	69.69(7)	F3	C16	F1	108.56(11)
C10	C9	Fe1	69.54(7)	F3	C16	F2	108.45(12)
C10	C9	C8	107.83(11)	F4	C17	S2	110.65(10)
C6	C10	Fe1	69.65(7)	F4	C17	F6	108.31(12)
C9	C10	Fe1	69.66(7)	F5	C17	S2	110.99(11)
C9	C10	C6	108.07(11)	F5	C17	F4	109.01(13)
C1	C11	N1	113.70(9)	F5	C17	F6	108.49(12)
C15	C14	N1	114.34(10)	F6	C17	S2	109.32(9)
C18	Fe2	C19	41.36(5)	O5	S3	N4	115.52(6)
C18	Fe2	C20	69.09(5)	O5	S3	C33	104.99(7)
C18	Fe2	C21	69.00(5)	O6	S3	O5	118.10(8)
C18	Fe2	C22	41.26(5)	O6	S3	N4	112.89(7)
C18	Fe2	C23	106.43(5)	O6	S3	C33	104.56(7)
C18	Fe2	C24	120.71(5)	N4	S3	C33	97.40(6)
C18	Fe2	C25	156.70(6)	O7	S4	O8	118.49(6)
C18	Fe2	C26	160.59(5)	O7	S4	N4	115.38(6)
C18	Fe2	C27	123.46(5)	O7	S4	C34	105.28(6)
C19	Fe2	C20	40.69(5)	O8	S4	N4	108.96(6)
C19	Fe2	C21	68.57(5)	O8	S4	C34	102.44(6)
C19	Fe2	C22	69.11(5)	N4	S4	C34	104.35(6)
C19	Fe2	C23	119.84(6)	S4	N4	S3	121.68(7)
C19	Fe2	C24	155.80(6)	F7	C33	S3	111.54(10)
C19	Fe2	C25	161.18(6)	F8	C33	S3	108.41(10)
C19	Fe2	C26	123.56(5)	F8	C33	F7	108.16(11)
C19	Fe2	C27	106.04(5)	F9	C33	S3	112.06(10)
C20	Fe2	C21	40.59(5)	F9	C33	F7	108.03(13)
C22	Fe2	C20	68.65(5)	F9	C33	F8	108.54(12)

C22	Fe2	C21	40.69(5)	F10	C34	S4	110.34(9)
C22	Fe2	C24	108.34(5)	F10	C34	F12	108.15(11)
C23	Fe2	C20	155.13(6)	F11	C34	S4	112.22(9)
C23	Fe2	C21	162.59(6)	F11	C34	F10	108.48(11)
C23	Fe2	C22	125.01(5)	F11	C34	F12	108.26(11)
C23	Fe2	C24	40.62(6)	F12	C34	S4	109.28(9)
